# Supplementary material for: Embedding of HIV Egress within Cortical F-Actin
Source: Pathogens. 2022 Jan 3;11(1):56. doi: 10.3390/pathogens11010056 (PMC8777837; doi:10.3390/pathogens11010056)
Supplement: Supplementary file 1 [file pathogens-11-00056-s001.zip › pathogens-1483251-supplementary figures and movies.pdf]

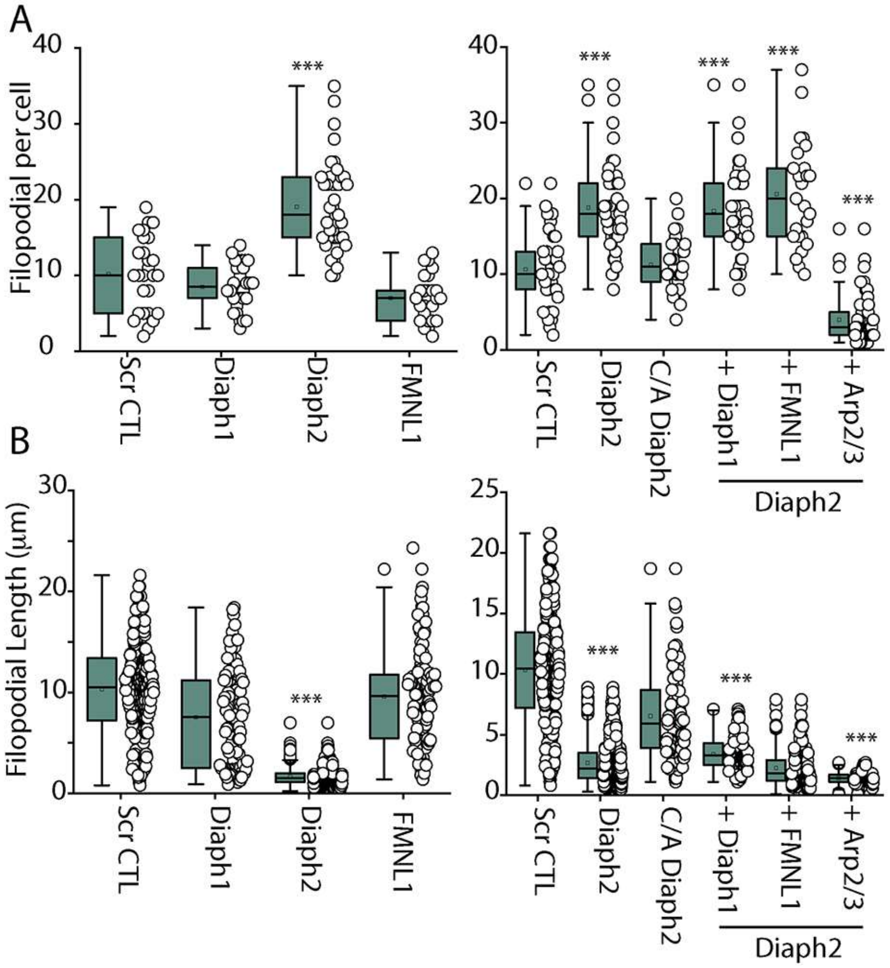

Figure S1. Filopodial network disruption as a result of formin and Arp2/3 depletion. Number of filopodia per cell were enumerated using live cell microscopy. In the left panel formins Diaph1, Diaph2 and FMNL1 are depleted using shRNA. Due to lack of detectable expression, other addition known formins were not tested. In the right panel, following depletion of Diaph2, cells were rescued with C/A Diaph2 or co-shRNA depleted (in addition to Diaph2 depletion) for the formins Diaph1, FMNL1 or the Arp2/3 complex. Note, only Arp2/3 + Diaph2 co-depletion decreases filopodial frequency. B. Filopodial lengths derived from the same cells observed in A. Note only Diaph2 depletion and Diaph2 & Arp2/3 co-depletion have significant impacts on filopodial lengths. Also note C/A Diaph2 can rescue filopodial lengths post Diaph2 depletion. Data represents pooled datasets from three independent experiments.***p<0.0001.

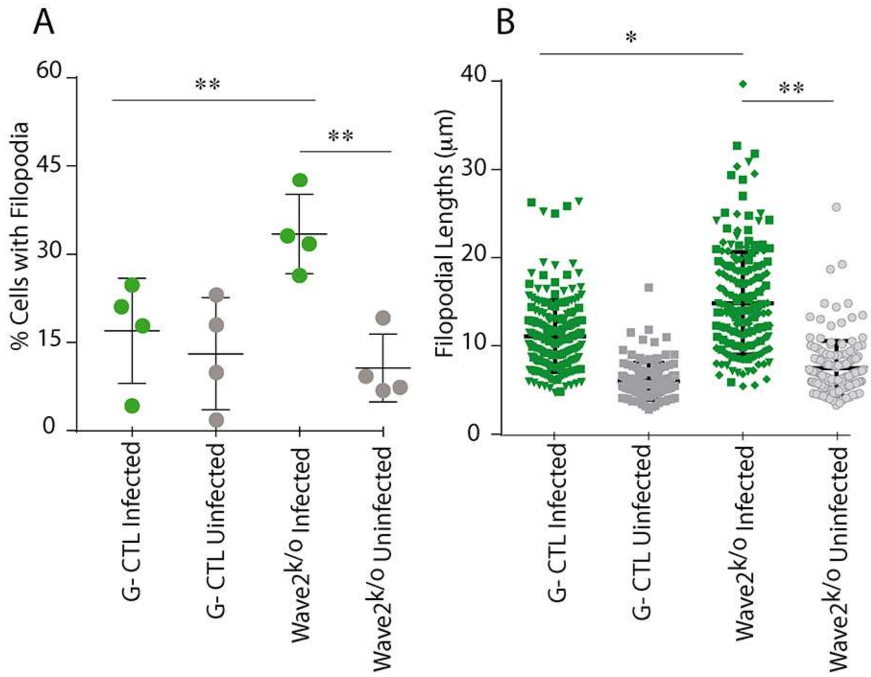

Figure S2. Filopodial frequencies and lengths relative to HIV infection

A. % Frequency of cells with filopodia. Infected (green) uninfected (grey). Each data point represents a single independent experiment B. Filopodial lengths in the presence of HIV infection. Accumulative filopodial lengths pooled from the 4 independent experiments outlined in A. *p<0.01, **p<0.001

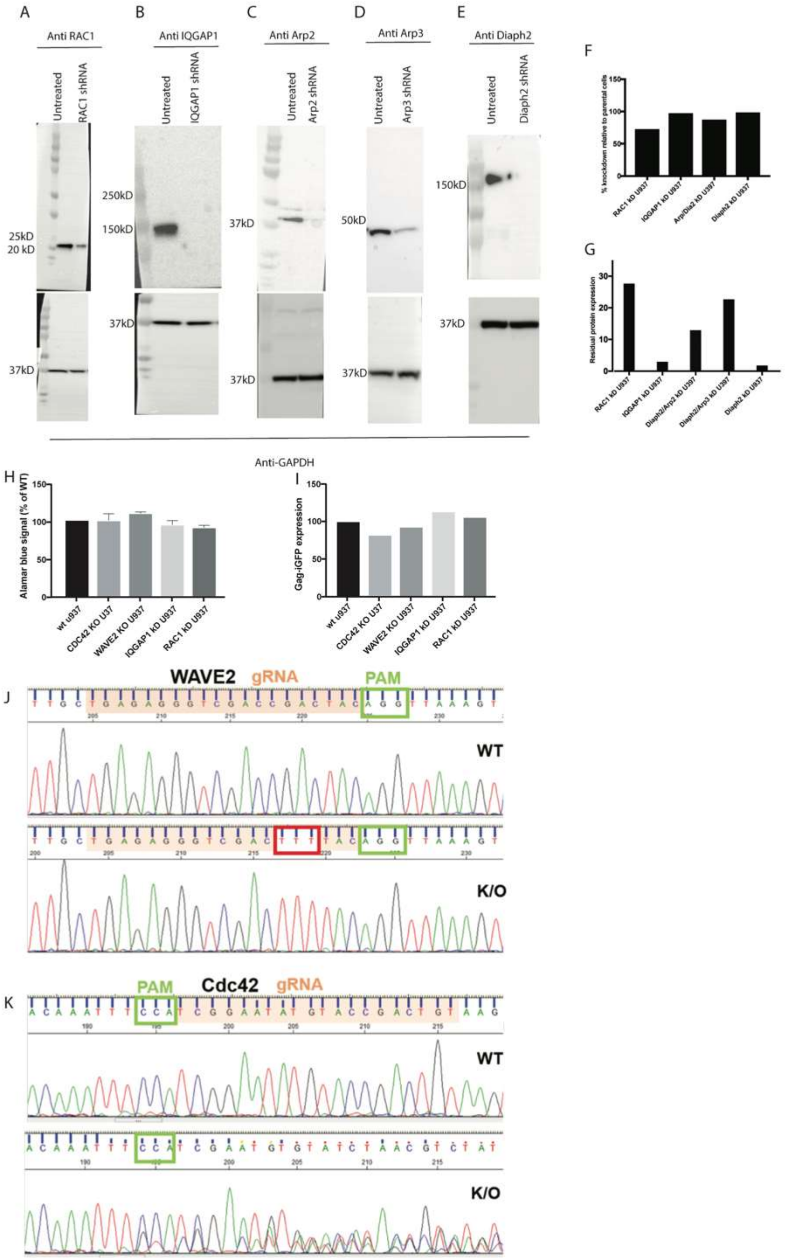

Figure S3. Analysis of clonal populations for shRNA knockdown & CRISPR/Cas9 gene-editing.

A-E Western blotting of cellular lysates using antigen specific antibodies to A. Rac1, B. IQGAP1, C. Arp2, D. Diaph2. In each case, GAPDH immunoblotting of the same transferred lysate is presented below each upper panel. F. Knockdown relative to untreated controls. G. Residual protein expression calculated by determining the band intensities using GeneTools software from Syngene followed by background correction and lane normalization of GAPDH. Remaining protein was then calculated by dividing the intensities of target bands by the lane normalization factor [17]. H. Metabolic activity (viability) of the main clonal cell lines used within this study. I. Relative Gag-iGFP expression within each clonal cell line. All cells were infected as outlined within quantitative cell to cell virus transfer assays. Cells were fixed and then mean fluorescence intensity quantified by flow cytometry.

Expression of Gag-iGFP is then expressed as % relative to the WT cell line control. In H and I, data is representative of a minimum of three independent control experiments. J-K U937 cells expressing Cas9 and a specific gRNA where clonally expanded, and clones were screened by genomic PCR amplification of the gRNA target site followed by DNA sequencing. Shown are representative examples for different gRNAs. In each case, the wildtype genome is shown above the mutated genome for comparison. The 20 bp region of the gRNA complementary to the host wildtype genome (protospacer) is highlighted in orange, whereas the protospacer adjacent motif (PAM sequence) is indicated by a green box (NGG in lead strand or CCN in complementary strand). **J.** Wave2$^{k/o}$ cell clone with three point mutations and 1 bp deletion in positions 14-17 of the protospacer (red box). Single chromatogram peaks throughout the amplicon indicate homozygous mutant alleles. The 1b deletion leads to a frameshift from residue 74, resulting in >70% loss of native protein sequence and a premature stop codon at residue 127 of 498. **K.** Cdc42$^{k/o}$ cell clone with heterozygous mutations, including at least 1 bp deletion per allele at positions 17-20 of the protospacer. Frameshift at these positions leads to >80% loss of native protein sequence.

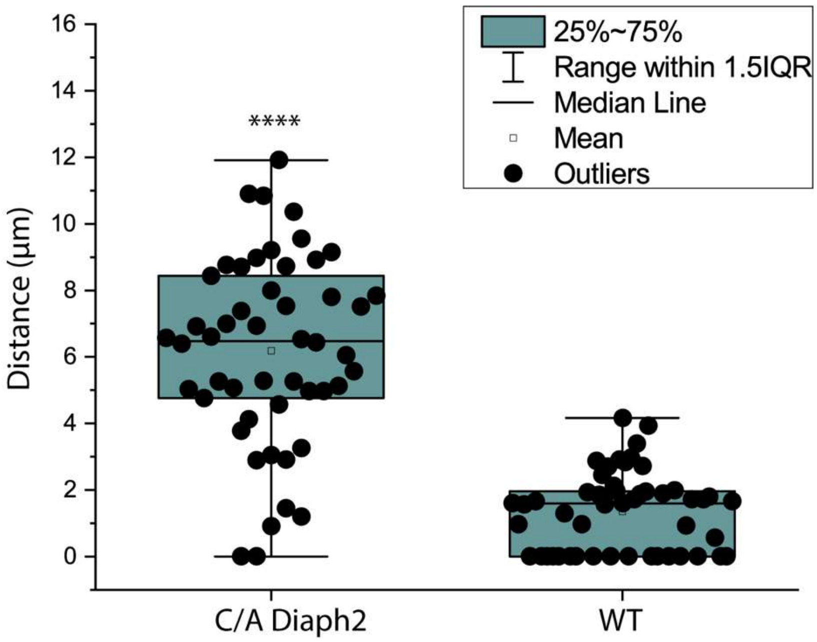

Figure S4. Arp2/3 antigen distance to filopodial tips in untreated and constitutively active Diaph2 expressing cells.

Distance (um) from the filopodial tip to the nearest detectable Arp2/3 site on the filopodial shaft.

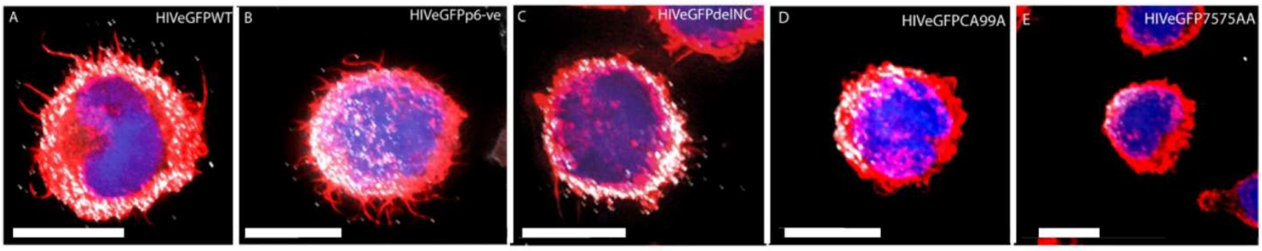

Figure S5. Localization of HIVGag in cells infected with Gag mutants. Diaph2^{-ve} were infected with A. HIViGFP WT. B. HIV Gag late domain mutant with p6 deleted. C. NC deletion mutant. D&E. HIV Gag curvature mutants D. P99A and E. EE75,76AA. Cells were fixed and counterstained with phalloidin Alexa-647 (red) and DAPI (blue). HIViGFP is shown in white. Scale bars are at 5µm.

<u>Supplementary movie files</u>

Movie S1. FIB-SEM imaging reveals extensive short filopodial networks in the absence of Diaph2.
Rotation of 3 dimensional rendering from accumulative *FIB-SEM* datasets derived from cells depleted of Diaph2. At the conclusion of the rotation, filopodial networks are highlighted individually in colour. Note the extensive curvature and sub-populations of short filopodia that have branching (i.e. Consistent with the action of Arp2/3).

Movie S2. FIB-SEM imaging reveals extensive lamellipodial networks in cells with co-depleted Diaph2 and Arp2/3.
Rotation of 3 dimensional rendering from accumulative *FIB-SEM* datasets derived from cells shRNA co-depleted of Diaph2 and Arp2/3. At the conclusion of the rotation, lamellipodial networks are highlighted individually in colour. For this dataset, we have used a HIV infected sample to highlight the enrichment of virions to the ridges of lamellipodial network (see timestamp 14s and the ridge of the green highlighted). Of note, HIV infection does not influence cortical F-Actin in this cellular clone and this image is representative of HIV negative and positive samples.

Movie S3. Diaph$^{C/A}$filopodia are straight, dynamic filopodia that exclude HIV.
Live imaging of HIViGFP^{+ve} (green) Diaph$^{C/A}$-mCherry (red) cells presented as an overlay with Differential Interference Contrast imaging to capture filopodial networks. Frame rate is presented in real-time at 0.841 frames per second.

Movie S4. Diaph$^{C/A}$ filopodia cannot be inhibited by HIV curvature mutants
Live imaging of Diaph^{-ve} HIViGFP$^{CA99A+ve}$(green) cells (left panel) and Diaph$^{C/A}$-mCherry (red), HIV$^{CA99A+ve}$ (green) cells presented as an overlay with Differential Interference Contrast imaging to capture filopodial networks. Frame rate is presented in real-time as in the figure legend to Movie 3. Note, in the left panel, the complete lack of any filopodial network, whereas in the right panel Diaph$^{C/A}$ can readily induce filopodia in the present of HIV$^{CA99A+ve}$.

Movie S5. HIV augmented filopodia in WAVE2$^{k/o}$ cells tether and then retract upon cell-cell conjugation. Tethering capacity of augmented filopodia is presented. Herein a thick filopodial structure at approximately 40 m in length is presented and targets cell that subsequently undergoes cell division. Note, that all filopodial activity ceases, once the infected donor cell has conjugated between the two recipient daughter cells. Movie is a time lapse speedup with each frame at 30 seconds intervals.

Movie S6. Filopodial networks in HIV infected WAVE2$^{k/o}$ cells collapse prior to cell-cell HIV transfer. Whilst extensive filopodial activity proceeds upon initial cell-cell contact, at the movie time point 31s, all filopodial activity ceases with retraction of all filopodia. Immediately following filopodial retraction, GFP delivery/release is firstly observed, followed shortly after by cell-cell fusion and syncytia formation. Movie is a time lapse speedup with each frame at 30 seconds intervals.
