# Supplementary material for: Embedding of HIV Egress within Cortical F-Actin
Source: Pathogens. 2022 Jan 3;11(1):56. doi: 10.3390/pathogens11010056 (PMC8777837; doi:10.3390/pathogens11010056)
Supplement: Supplementary file 1 [file pathogens-11-00056-s001.zip › pathogens-1483251-supplementary tables.pdf]

Supplementary tables

Short-Filopodia (Diaph^{-ve})		Lamellipodia (Diaph^{-ve} Arp2/3^{-ve})	
Tip vs PM[1]	0.028	Ridge vs PM	0.121
Tip vs shaft	0.026	Ridge vs face	0.13
Tip vs base	0.022	Ridge vs base	0.124
Shaft vs PM	0.539	Face vs PM	0.91
Base vs PM	0.153	Base vs PM	0.975
Base vs shaft	0.513	Base vs face	0.94

[1]PM = Plasma membrane

Table S1. Statistical summary of HIV bud frequencies

Table S2.

Plasmid constructs generated and used in this study

Plasmids	Description
HIViGFP	HIV carrying eGFP between MA and CA domains of Gag
HIViGFP^{ENV-ve}	HIV carrying eGFP between MA and CA domains of Gag but lacking a functional ENV due to presence of stop codons in ENV ORF
HIV^{NC-ve}	HIViGFP Gag mutant wherein the NC domain has been replaced with Leucine zipper domain of yeast transcription factor GCN4
HIV^{CA7576AA}	HIViGFP Gag mutant carrying point mutations EE7576AA in the CA domain of Gag
HIV^{CA99A}	HIViGFP Gag mutant carrying point mutations P99A in the CA domain of Gag
HIV^{p6-ve}	HIViGFP Gag mutant with deleted p6 domain
pHIVNL43IRESeGFP	HIV NL34 expressing eGFP in the Nef ORF followed by an IRES element and an intact HIV Nef ORF.
pLVXdeltaDADmcherry	Lentiviral plasmid expressing the constitutively active form of Diaph2

Table S3. shRNA sequences used in this study

Name	Target sequence	Sequence ID	Source
Arp2a	GTGGGTAAATCTGAGTTTA	N/A	Gomez et al; 2007

Arp2b	CTGTGTTACGTGGGATATA	N/A	Gomez et al; 2007
Arp3a	GAAGAGAGCGAAAGTGATT	N/A	Gomez et al; 2007
Arp3b	GAGCAAGTGATCTTTAAATA	N/A	Gomez et al; 2007
Diaph1_1	GCATGCCCTATCAAGAGATTA	TRCN0000118681	TRC, Broad Institute
Diaph1_2	GCCTCCTTATTGGACATTCTT	TRCN0000118679	TRC, Broad Institute
Diaph3_1	CCAGATTTGTTATCAGTGCAA	TRCN0000152084	TRC, Broad Institute
Diaph3_2	GCATGACAAGTTTGTGACAAA	TRCN0000150850	TRC, Broad Institute
Diaph2_1	GCATCAATGGAACAACAAAtt	sc-35192-SHA	Santa Cruz
Diaph2_2	GGAACGTGACATCAAGAAAtt	sc-35192-SHB	Santa Cruz
Diaph2_3	GGAATCCAGTGGTAAATCAtt	sc-35192-SHC	Santa Cruz
FMNL1_1	CCCTCTTTAGCCGCTTCATTA	TRCN0000008289	TRC, Broad Institute
FMNL1_2	GAGCGGTTTCAAGTCAAGAAT	TRCN0000008287	TRC, Broad Institute
RAC1_1	CCCTACTGTCTTTGACAATTA	TRCN0000004873	TRC, Broad Institute
RAC1_2	CGCAAACAGATGTGTTCTTAA	TRCN0000004871	TRC, Broad Institute
IQGAP1_1	GCCCACATTGTGCCTTTATTT	TRCN0000047483	TRC, Broad Institute
IQGAP1_2	GCATCCACTTACCAGGATATA	TRCN0000047487	TRC, Broad Institute

Table S4. CRISPR gRNA used in this study

Name	gRNA sequence (5'-3') with target region	Target gene (NCBI GeneID)
Diaph2 gRNA _Oligo 1	caccgGAACCGGGCCGCCAATGAAG	Diaph2 (Gene ID: 1730)
Diaph2 gRNA _Oligo2	aaacCTTCATTGGCGGCCCGGTTCc	"-"
Diaph2 Surveyor Fwd	CCCGAGGTGCTTTCTCAGTT	"-"
Diaph2 Surveyor Rvs	GGTTTGCGCCCACAAGTTAG	"-"
Cdc42 gRNA _Oligo 1	caccgGACAGTCGGTACATATTCCGA	Cdc42 (Gene ID: 998)
Cdc42 gRNA _Oligo2	aaacTCGGAATATGTACCGACTGTCc	"-"
Cdc42 Surveyor Fwd	TTGCTCTGAGTGCCTGAACC	"-"
Cdc42 Surveyor Rvs	AGTTTTGCCTCCCAGCACTT	"-"
IQGAP1 gRNA _Oligo 1	caccgGTGGGGTCTACCTTGCCAAAC	IQGAP1 (Gene ID:8826)
IQGAP1 gRNA _Oligo2	aaacGTTTGGCAAGGTAGACCCCACc	"-"
IQGAP1 Surveyor Fwd	CCTCTCTCCTTTGGTGCAGG	"-"
IQGAP1 Surveyor Rvs	GAGGCGGGCTTTCCAGATAA	"-"
Rac1 gRNA _Oligo 1	caccgGTGGTTGTGTAACTGATCAGT	Rac1 (Gene ID:5879)
Rac1 gRNA _Oligo2	aaacACTGATCAGTTACACAACCACc	"-"
Rac1 Surveyor Fwd	GGTACCAATGTGTATGTGGTGA	"-"
Rac1 Surveyor Rvs	CCTCACAGACAGTTAAAGTTTGC	"-"
WAVE2 gRNA _Oligo 1	caccgGTGAGAGGGTCGACCGACTAC	WAVE2 (GeneID:10163)
WAVE2 gRNA _Oligo2	aaacGTAGTCGGTCGACCCTCTCACc	"-"
WAVE2 Surveyor Fwd	GCAGTCATCTGCATATAGCCCA	"-"
WAVE2 Surveyor Rvs	GCTCAGCAGAGCCTGCAAAT	"-"

Table S5. List of selected proteins detected in virion preparations from Diaph2^{+ve}Arp^{+ve} Diaph2^{-ve}Arp^{+ve} cells and Diaph2^{-ve}Arp^{-ve} cells.

**Protein name**[1]	**Gene name**	**Function**	**Mol Wt (kDa)**	**Number of peptides**[2]		
				Diaph2^{+ve}Arp^{+ve} cells	Diaph2^{-ve}Arp^{+ve} cells	Diaph2^{-ve}Arp^{-ve} cells
Cadherin-2	CDH2	Cell to cell adhesion	99.809	2	2	0
Cadherin-6	CDH6	Cell to cell adhesion	88.309	4	1	1
Cadherin-13	CDH13	Cell to cell adhesion	78.287	3	3	2
Integrin alpha-X (CD11c)	ITGAX	Cell to cell adhesion	127.829	4	4	0
Integrin alpha-M	ITGAM	Cell to cell adhesion	127.179	9	7	12
Integrin alpha-L (CD11a)	ITGAL	Cell to cell adhesion	128.77	6	4	5
Integrin alpha-4	ITGA4	Cell to cell adhesion	114.90	6	7	1
Integrin beta	ITGB1	Cell to cell adhesion	88.41	10	17	9
Integrin alpha-5	ITGA5	Cell to cell adhesion	114.536	9	6	11
Integrin alpha-6	ITGA6	Cell to cell adhesion	126.60	9	8	9
CD44 antigen	CD44	Cell to cell adhesion	81.538	8	7	4
CD97 antigen	CD97	Cell to cell adhesion	91.868	7	5	0
Intercellular adhesion molecule 1	ICAM1	Cell to cell adhesion	57.825	7	5	5
Platelet endothelial cell adhesion molecule	PECAM1	Cell to cell adhesion	82.522	13	13	2

Protein name[1]	Gene name	Function	Mol Wt (kDa)	Number of peptides[2]		
				Diaph2^{+ve}Arp^{+ve} cells	Diaph2^{-ve}Arp^{+ve} cells	Diaph2^{-ve}Arp^{-ve} cells
C-type lectin domain family 2 member B	CLEC2B	Cell to cell adhesion	17.307	3	3	2
Ezrin	EZR	Connects actin cytoskeleton to PM	69.413	4	1	1
Talin-1	TLN1	Links integrins to actin cytoskeleton	269.967	22	13	12
Alpha-actinin-1	ACTN1	Connects actin cytoskeleton to intracellular structures	103.058	7	7	6
FilaminA	FLNA	Links actin to TM proteins	280.739	11	9	13
Moesin	MSN	Connects actin cytoskeleton to PM	67.82	32	20	38
Vinculin	VCL	Links integrins to actin cytoskeleton	123.799	36	31	23
Actin-related protein 2/3 complex subunit 3	ARPC3	Actin nucleation	20.547	2	3	0

Protein name[1]	Gene name	Function	Mol Wt (kDa)	Number of peptides[2]		
				Diaph2^{+ve}Arp^{+ve} cells	Diaph2^{-ve}Arp^{+ve} cells	Diaph2^{-ve}Arp^{-ve} cells
Actin-related protein 2/3 complex subunit 2	ARPC2	Actin nucleation	34.333	2	3	0
Actin related protein 2	ACTR2	Actin nucleation	44.761	7	5	1
F-actin-capping protein subunit beta	CAPZB	Actin regulation	31.35	4	4	2
F-actin-capping protein subunit alpha-1	CAPZA1	Actin regulation	32.923	4	5	0
Profilin 1	PFN1	Actin regulation	15.054	9	7	6
Macrophage-capping protein	CAPG	Actin regulation	36.857	11	11	7
Ras GTPase-activating-like protein IQGAP1	IQGAP1	Actin regulation	189.252	17	15	13
Cell division control protein 42 homolog	CDC42	Actin regulation	21.258	2	3	4
Ras-related C3 botulinum toxin substrate 1	RAC1	Actin regulation	21.45	2	2	2
Transforming protein RhoA	RHOA	Actin regulation	21.768	3	3	2

Protein name[1]	Gene name	Function	Mol Wt (kDa)	Number of peptides[2]		
				Diaph2^{+ve}Arp^{+ve} cells	Diaph2^{-ve}Arp^{+ve} cells	Diaph2^{-ve}Arp^{-ve} cells
Rac GTPase-activating protein 1	RACGAP1	Regulation of RhoGTPases	71.026	9	10	2
Rho GDP-dissociation inhibitor 2	ARHGDIB	Regulation of RhoGTPases	22.988	5	4	4

[1]Alternative names are given in parentheses
[2]Proteins with 2 or more peptides were considered valid hits

Table S6. Fold change in the levels of cellular proteins detected in virion preparations from Diaph2^{-ve}Arp^{+ve} cells and Diaph2^{-ve}Arp^{-ve} cells relative to Diaph2^{+ve}Arp^{+ve} cells.

Protein name[1]	Fold Change (relative to Diaph2^{+ve}Arp^{+ve})[2]	
	Diaph2^{-ve}Arp^{+ve}	Diaph2^{-ve}Arp^{-ve}
Villin-1	ND	ND
Cadherin-2	ND	ND
Cadherin-6	ND	ND
Junction plakoglobin	ND	ND
Ezrin	0.426869319	0.775834415
Integrin alpha-X	0.594352604	ND
F-actin-capping protein subunit beta	0.664438528	0.997147476
Integrin alpha-M (CD11b)	0.70278725	0.318636027
Profilin	0.729396153	0.423568972
Integrin alpha-L (CD11a)	0.775366489	0.641602058
Actin-related protein 2/3 complex subunit 3	0.78379315	ND
Macrophage-capping protein	0.82027724	0.472195358
CD44 antigen	0.827739842	0.850743377
Intercellular adhesion molecule 1	0.865094017	1.282666667
Actin-related protein 2/3 complex subunit 2	0.886295503	ND
Rac GTPase-activating protein 1	0.891894534	0.144910863
CD97 antigen	0.89546113	ND

Cadherin-13	0.907164703	1.087725548
Talin-1	0.917354722	0.906555529
Ras GTPase-activating-like protein IQGAP1	0.921946106	1.03400317
Platelet endothelial cell adhesion molecule	0.941177775	0.137107002
Alpha-actinin-1	0.943789847	0.960939391
Integrin alpha-4	0.954788728	ND
Integrin beta	0.990104053	1.023757549
FilaminA	1.033951235	1.148007379
Pyruvate kinase	1.084727918	1.312756289
Transforming growth factor-beta-induced protein ig-h3	1.098560722	1.004211713
F-actin-capping protein subunit alpha-1	1.134261145	ND

Protein name[1]	Fold Change (relative to Diaph2^{+ve}Arp^{+ve})[2]	
	**Diaph2^{-ve}Arp^{+ve}**	**Diaph2^{-ve}Arp^{-ve}**
Ras-related C3 botulinum toxin substrate 1 (Rac1)	1.147066448	1.210101097
Moesin	1.20691883	1.678873488
Vinculin	1.229865447	0.933886541
Rho GDP-dissociation inhibitor 2	1.32454502	0.850584198
Guanine nucleotide-binding protein G(i) subunit alpha-2	1.331356723	0.952451546
Integrin alpha-6	1.338333706	1.306515412
C-type lectin domain family 2 member B	1.494409642	1.392593767
ARP2 actin-related protein 2 homolog	1.564918357	ND
Cell division control protein 42 homolog (Cdc42)	1.681728101	1.028193949
Transforming protein RhoA	1.739662389	0.991720712
Integrin alpha-5	2.195967025	1.822671216

[1]Alternative names are given in parentheses

[2]Fold change was calculated as the ratio of MaxQuant LFQ intensities of peptides observed in virion preparations from mutant cell lines over preparations from Diaph2^{+ve}Arp^{+ve} cells
